# Subgingival Microbiome in Young Adult Cigarette Smokers, E-Cigarette Users, and Non-Smokers

**DOI:** 10.3390/medicina62081544

**Published:** 2026-08-11

**Authors:** Mihaela Mociu, Cristina Gabriela Pușcașu, Steliana Gabriela Buștiuc, Cristina Bartok-Nicolae, Gheorghe Raftu, Raluca Briceag, Aureliana Caraiane

**Affiliations:** Department of Dentistry, Faculty of Dentistry, “Ovidius” University of Constanța, 900527 Constanta, Romania; mihaelabacula@gmail.com (M.M.); dr.cristina_nicolae@yahoo.com (C.B.-N.); gheorgheraftu@yahoo.com (G.R.); ralubriceag@yahoo.com (R.B.); drcaraiane@yahoo.com (A.C.)

**Keywords:** cigarette smoking, electronic nicotine delivery systems, microbiota, vaping, young adult

## Abstract

*Background and Objectives:* Cigarette smoking is a recognized risk factor for periodontal disease and subgingival dysbiosis; however, the impact of electronic cigarettes on the subgingival microbiome in young adults is still not sufficiently investigated. This study aimed to characterize and compare the carriage and bacterial load of 20 subgingival species among young adult cigarette smokers, e-cigarette users, and non-smokers, and to evaluate whether e-cigarette use is linked to a periodontopathogen-enriched profile similar to that of smoking. *Materials and Methods:* In this exploratory cross-sectional study, fifty young adults were divided into three groups: cigarette smokers (*n* = 18), e-cigarette users (*n* = 16), and non-smokers (*n* = 16). Subgingival specimens were examined using real-time Polymerase Chain Reaction (PCR) (Perio panel; ADD Laboral B.V., Malden, The Netherlands) for 20 bacterial species. Carriage was analyzed across groups utilizing Fisher’s exact test with Cramér’s V as the effect size, while bacterial load was assessed using the Kruskal–Wallis test accompanied by Dwass–Steel–Critchlow–Fligner post hoc comparisons (jamovi; significance threshold established at *p* < 0.05). *Results:* Red-complex pathogens were more prevalent and present in greater quantities in smokers and e-cigarette groups compared to non-smokers. *Porphyromonas gingivalis* prevalence was seen in smokers (94.4%), vapers (75.0%), and non-smokers (56.2%; *p* = 0.028), whereas *Tannerella forsythia* was found in 100% of smokers, 87.5% of vape users, and 68.8% of the non-smokers (*p* = 0.022). *Aggregatibacter actinomycetemcomitans* was not present in non-smokers but was identified in 37.5% of vapers and 16.7% of smokers (*p* = 0.018), whereas *Eubacterium nodatum* was significantly elevated in tobacco users (*p* = 0.012; Cramér’s V = 0.449). In contrast, *Prevotella nigrescens* was predominant among non-smokers (87.5%; *p* = 0.022). *Conclusions:* In young adults, the red-complex bacteria—*Porphyromonas gingivalis*, *Tannerella forsythia*, and *Treponema denticola*—were found more frequently and in larger quantities among smokers and vapers as compared to non-smokers.

## 1. Introduction

The human oral cavity, characterized by its moist and organic-rich environment, serves as an ecosystem where a vast array of microorganisms exist and flourish [1]. Periodontal health is characterized by the absence of clinically detectable inflammation, supported by a baseline degree of immune surveillance consistent with gingival health and tissue homeostasis. Clinical gingival health can occur both on an intact periodontium and on a reduced periodontium, either in individuals with no history of periodontitis or in periodontitis patients who are currently stable [2]. In a healthy periodontium and in cases of gingivitis, competition among inter-microbial species appears to self-regulate, resulting in microbial equilibrium [3]. Recent notions indicate that keystone pathogens might affect tissue homeostasis and alter the composition of the commensal microbiota, resulting in host immunological regulation and dysbiosis, which contribute to periodontitis [4]. Periodontal pathogens can enter the bloodstream or lymphatic system, contributing to systemic inflammatory and autoimmune illnesses, such as cardiovascular disease, Alzheimer’s disease, colorectal cancer, metabolic disorders, and problems during pregnancy [5]. *Porphyromonas gingivalis* is recognized for its critical role in the formation of dysbiotic microbial communities, capable of disrupting host-microbial balance and triggering inflammatory responses through interactions with other oral bacteria [6]. Research indicates that smoking may cause oral dysbacteriosis [7] and negatively affect the occurrence and progression of periodontitis [8]. Chronic tobacco consumption, especially smoking, has consistently been linked to the inhibition of angiogenesis in both healthy individuals and patients with periodontal disease [9]. Dysbiosis in smokers persisted significantly even after the resolution of experimentally-induced gingivitis and the recovery of clinical symptoms of periodontitis with non-surgical periodontal treatment, remaining evident for almost three months post-therapy [10]. Although smokers exhibit heightened vulnerability to diseases, overt mouth inflammation is diminished, creating a diagnostic dilemma [11]. An analysis has confirmed a significant influence of smoking on the core salivary microbiome, highlighting differences in relative abundance percentages between smokers and non-smokers [12]. Research indicates that the outcomes of periodontal treatment are less favorable in smokers compared to non-smokers [13].

E-cigarettes were previously perceived as a potential aid for smoking cessation, particularly among adults [14]. The rising prevalence of e-cigarettes has been observed to disturb microbial equilibrium in the mouth [15,16], and also to elevate opportunistic pathogens [15]. E-cigarette users may have elevated inflammation and increased vulnerability to upper respiratory infections, like those related to tobacco smokers [17].

The heterogeneity expected across study designs, outcome measures, and sampling approaches, in a recent systematic review on the impact of electronic cigarettes, highlights both the importance of interpreting results cautiously and the need for greater methodological standardization in future studies [18].

Because studies differ considerably on nicotine concentration, flavoring substances, duration of e-cigarette exposure, and patient inclusion and exclusion criteria, not all were specifically designed to evaluate oral health effects. This underscores the need for larger, longitudinal studies conducted on more homogeneous cohorts of e-cigarette users [17].

Given these limitations, there is a clear need for studies that directly and simultaneously compare cigarette smokers, e-cigarette users, and non-smokers within a single, standardized methodological framework, using objective microbiological quantification rather than self-reported oral health measures alone. Investigating a periodontally healthy population, using a validated PCR-based approach and a duration-matched exposure criterion across groups, allows for a more controlled assessment of whether e-cigarette use is associated with a distinct, intermediate, or comparable subgingival bacterial risk profile relative to conventional cigarette smoking.

This study concentrated on young adults, where e-cigarette use is increasingly prevalent, and the eventual periodontal damage may still be in an early, potentially reversible phase. The primary objective of this study was to define and compare the subgingival microbiome, represented by the presence and bacterial load of 20 selected species, among young adult cigarette smokers, e-cigarette users, and non-smokers.

## 2. Materials and Methods

### 2.1. Study Design

This was an exploratory, observational, analytical, cross-sectional, comparative study involving three parallel groups (cigarette smokers, e-cigarette users, and non-smokers), conducted to evaluate differences in subgingival bacterial load and periodontal clinical parameters among the study groups. This study was performed at the School of Dental Medicine, Ovidius University of Constanta, Constanta, Romania. The Ethics Committee of the Faculty of Dental Medicine at the University of Ovidius granted approval for this study’s approach (15089/9 December 2025). All participants joined voluntarily and agreed to complete their informed consent forms to engage in this study. Their identities were kept confidential. This research was conducted in compliance with the World Health Organization’s Declaration of Helsinki [19].

### 2.2. Subjects

The inclusion criteria were good general health and absence of any lesions below, at, or above the level of the oral mucosa. The target population was young adults aged 18 to 30 years of both genders selected according to predefined smoking/vaping exposure thresholds. Cigarette smokers were defined as individuals who reported smoking a minimum of five conventional cigarettes per day for at least three consecutive years. E-cigarette users were defined as individuals who reported consuming a minimum of 0.4 mL of e-liquid per day for at least three consecutive years. The duration and intensity-based thresholds were applied consistently across both exposure groups to ensure a comparable minimum level of chronic exposure prior to enrollment. The participants needed to have good systemic health, with the groups possessing whole anterior teeth in the maxillary or mandibular arch. The control group consisted of non-smokers from the same age group, also without systemic pathology. Participants were required to be periodontally healthy, as determined by clinical examination, with bleeding on probing (BOP) present at fewer than 10% of sites and probing depths not exceeding 3 mm at any site, confirmed through clinical periodontal examination. This clinical periodontal health status constituted an inclusion criterion for participation in this study.

Participants utilizing daily chlorhexidine-based mouth antiseptics; pregnant individuals; patients exhibiting systemic diseases; individuals on any pharmacological therapy or medication; subjects with a previous record of periodontal treatment; those with anterior teeth with any composite restorations, prosthetics, crowns, or veneers; individuals with recent whitening treatment; individuals who had professional cleaning within the last 24 weeks prior to screening; individuals who used antibiotics in the last 6 months; those with tooth jewelry or orthodontic appliances on their vestibular teeth; patients with unsatisfactory oral hygiene; and those with an OHI-S index greater than 3 were also excluded from this study.

Participants were recruited between February and June 2026. No formal sample size calculation was performed; so, the final sample size was determined by the number of eligible participants meeting the inclusion criteria assessed during the recruitment period.

**Figure 1:**
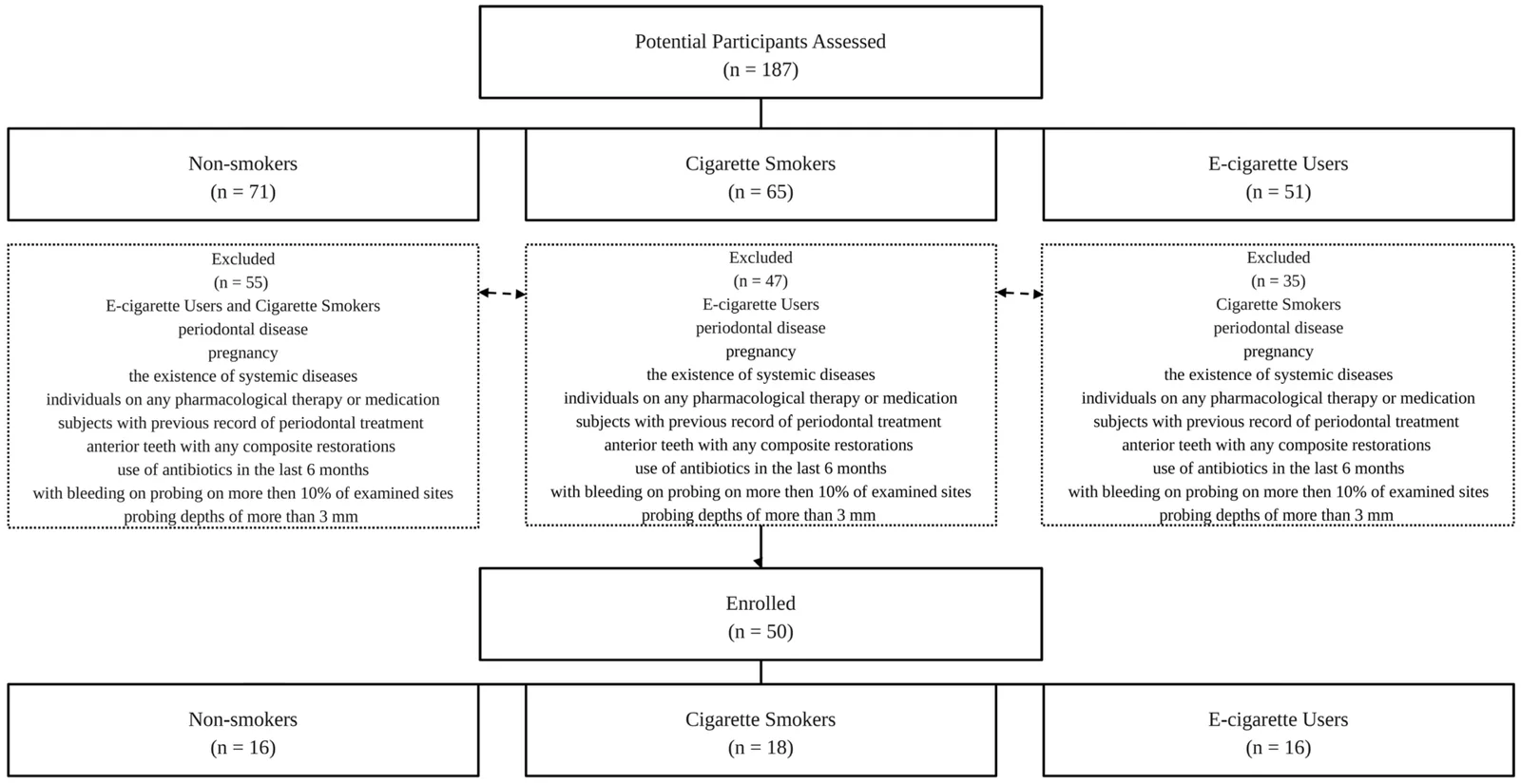
Study flow.

Fifty individuals met the inclusion criteria for this study (Figure 1). They were directed to refrain from brushing their teeth, smoking, consuming food, or drinking anything other than water for two hours before the clinical examination and bacterial sampling.

### 2.3. Anamnesis and Clinical Examination

This study is part of a more complex investigation aimed to assist the relation between smoking, vaping, and non-smoking on the periodontal status and color changes in the teeth in a young population. The processing protocol for each patient involved collecting anamnestic data, doing a clinical examination of the oral cavity, and obtaining paper-point sample swabs from the gingival sulcus. The participants also completed questionnaires for this complex investigation. The clinical examination encompassed an assessment of all teeth. To ensure standardization, all assessments were performed by the same examiner in a single examination room under consistent ambient-lighting conditions. Soft-tissue retraction was achieved using the OptraGate system (Ivoclar Vivadent, Schaan, Liechtenstein). The clinical examination was performed using a PCP-15 mm graduated manual periodontal probe (Hu-Friedy, Chicago, IL, USA). The following clinical parameters were assessed: probing depth (PD), The Simplified Oral Hygiene Index (OHI-S) [20], which combines the Debris Index and the Calculus Index scored on six index-tooth surfaces, measuring the depth of the periodontal pocket if values are greater than 3 mm and bleeding on probing (BoP).

### 2.4. Microbiological Sampling and Analyses

Samples for microbiological analysis were obtained with 4 or 5 paper points from areas that BoP was recorded clinically. All participants first underwent a clinical periodontal examination, during which BoP, OHI-S, and PD were recorded at baseline. Approximately one week later, eligible participants were recalled for microbiological sampling. Subgingival plaque samples for PCR analysis were collected from the sites that had exhibited bleeding on probing at the baseline clinical examination.

This approach was intended to maximize the likelihood of detecting periodontal pathogens that may be preferentially localized to sites of early even subclinical inflammation, thereby reducing the risk of false-negative findings that could result from sampling clinically uninvolved sites randomly chosen.

The subgingival microbiota was analyzed by quantitative real-time PCR (qPCR) using the Perio 20 panel (ADD Laboral B.V., Malden, The Netherlands), which detects 20 periodontal bacterial species. The teeth were isolated in a dry working area by placing cotton rolls in the openings of the major salivary glands and positioning the saliva ejector beneath the mouth to prevent saliva contamination of the paper points, followed by additional drying using compressed air. The supragingival biofilm was removed using a sterile cotton swab. The paper point was inserted into the gingival sulcus for a range of 20–30 s and then placed into a sterile empty tube and sent to the laboratory for interpretation.

### 2.5. Statistical Data Analysis

This study should be interpreted as an exploratory investigation. The collected data were processed and analyzed using Microsoft Excel (Microsoft Corporation, Washington, DC, USA) and mean (average) and standard deviation were calculated. The statistical analyses were performed using jamovi (version 2.7.33; the jamovi project, Sydney, Australia). The study population contained 50 young adults allocated to three groups: cigarette smokers (*n* = 18), e-cigarette users (*n* = 16), and non-smokers (*n* = 16). Categorical data, including the carriage (presence/absence) of each of the 20 bacterial species, were expressed as absolute numbers and percentages. Because the bacterial load values were continuous, but did not follow a normal distribution, non-parametric methods were applied. Differences in bacterial load among the three groups were assessed using the Kruskal–Wallis test, and statistically significant results were further analyzed with Dwass–Steel–Critchlow–Fligner (DSCF) pairwise comparisons. Differences in the detection frequency (prevalence) of each species among the groups were evaluated using Fisher’s exact test; the strength of the association between group and species carriage was quantified using Cramér’s V. The *p*-value < 0.05 was considered statistically significant.

## 3. Results

Fifty patients (in the 18–30-year-old age group interval) were included in this study, out of which 18 were smokers, 16 were vape users, and 16 were non-smokers.

The mean OHI-S score was 0.5362 ± 0.2689 in the non-smoker group (*n* = 16), corresponding to good oral hygiene (<1.2). In contrast, the mean OHI-S score was 1.6422 ± 0.4064 for cigarette smokers (*n* = 18) and 1.5681 ± 0.3955 for e-cigarette users (*n* = 16), with both values falling within the fair oral hygiene range (1.3–3.0).

**Figure 2:**
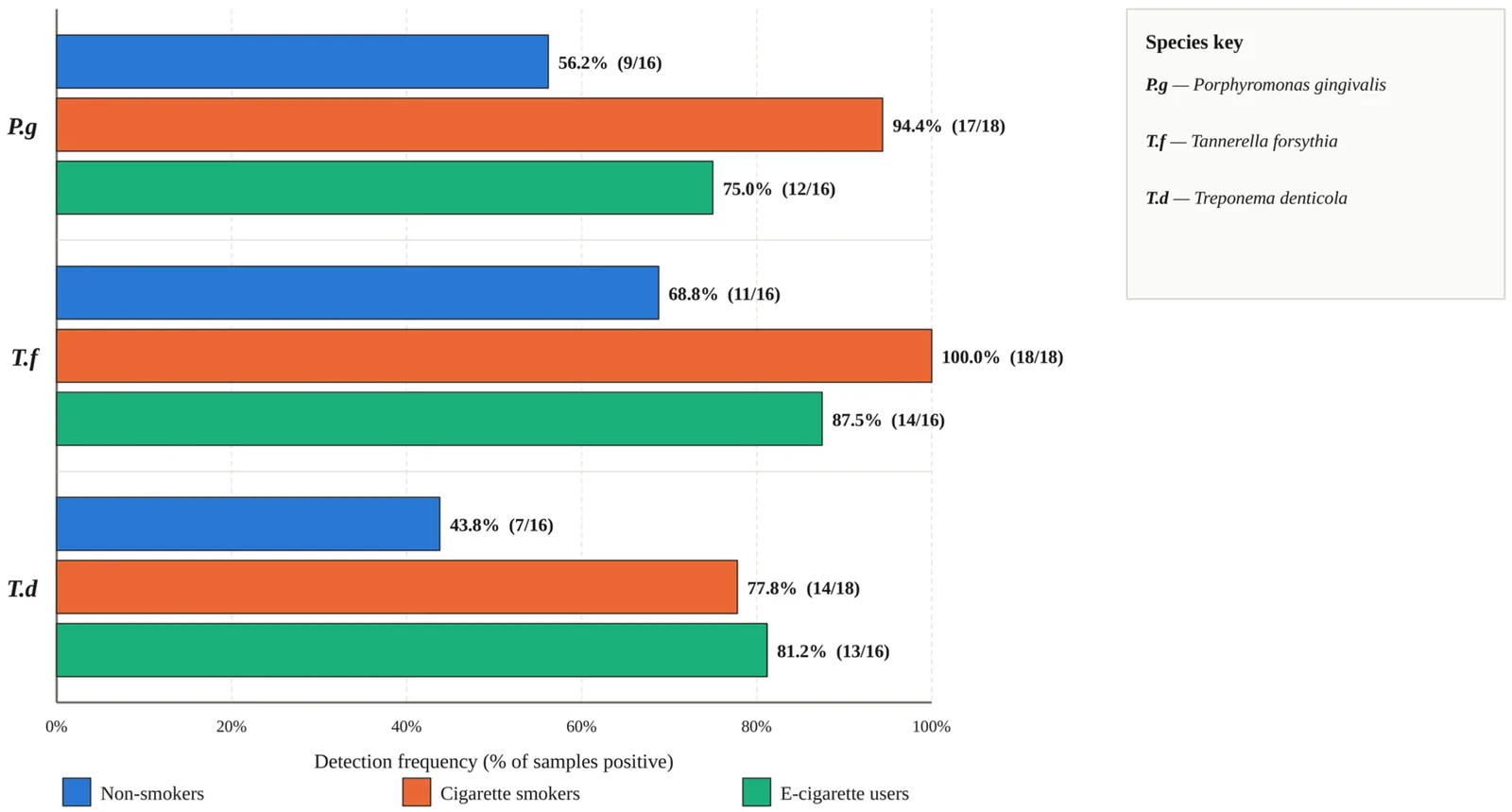
Detection frequency of red-complex bacteria by study group.

The carriage rates of the 20 subgingival species in the three groups, together with the corresponding Fisher’s exact test and Cramér’s V values, are presented in Table 1, while the differences in bacterial load between groups are shown in Table 2 and Figure 2.

A consistent pattern was observed for the periodontal pathogens of the red complex, which were carried more frequently and at higher loads in the smoker and vape-users groups than in non-smokers. *Porphyromonas gingivalis* was detected in 94.4% of cigarette smokers and 75.0% of e-cigarette users, but in only 56.2% of non-smokers (Fisher’s exact *p* = 0.028; Cramér’s V = 0.368), and its load differed significantly among the groups (χ^2^ = 15.581, *p* < 0.001). *Tannerella forsythia* followed the same trend, being present in all smokers (100%) and in 87.5% of vapers versus 68.8% of non-smokers (*p* = 0.022; V = 0.372), with a significant difference in load (χ^2^ = 20.062, *p* < 0.001). *Treponema denticola* was carried by 77.8% of smokers and 81.2% of vapers, with non-smokers having just 43.8%; although the difference in carriage was only borderline (*p* = 0.056), the difference in load was significant (χ^2^ = 11.259, *p* = 0.004).

*Aggregatibacter actinomycetemcomitans* showed a distinctive distribution: it was absent in all non-smokers (0%) but present in 37.5% of e-cigarette users and 16.7% of smokers (*p* = 0.018; V = 0.391; load χ^2^ = 7.745, *p* = 0.021), being most frequent in the vaping group.

*Eubacterium nodatum* was likewise more prevalent in tobacco users (83.3% of smokers and 81.2% of vapers) than in non-smokers (37.5%) (*p* = 0.012; V = 0.449; load χ^2^ = 10.741, *p* = 0.005). A significantly higher load, although without a significant difference in carriage, was also found for *Fusobacterium nucleatum* (χ^2^ = 10.648, *p* = 0.005), *Peptostreptococcus micros* (χ^2^ = 7.42, *p* = 0.024), *Campylobacter rectus/showae* (χ^2^ = 10.589, *p* = 0.005), and *Campylobacter concisus* (χ^2^ = 6.865, *p* = 0.032). *Campylobacter gracilis* was more frequent in smokers (72.2%) than in vapers (50.0%) or non-smokers (31.3%) (*p* = 0.063; load χ^2^ = 6.042, *p* = 0.049).

Two species showed the opposite pattern, being more common in non-smokers. *Prevotella nigrescens* was carried by 87.5% of non-smokers but by only 50.0% of smokers and 43.8% of vapers (*p* = 0.022; V = 0.389). *Capnocytophaga* species were also more prevalent in non-smokers (56.2%) than in smokers (27.8%) or vapers (18.8%), although this difference did not reach significance (*p* = 0.086). The early-colonizing commensal *Streptococcus gordonii* tended to be depleted in smokers, and was detected in only 38.9% of them compared with 75.0% of non-smokers and 68.8% of vapers (*p* = 0.067; load χ^2^ = 5.908, *p* = 0.052).

After analyzing the microbiota of cigarette smokers and non-smokers, the contrast yielded the highest number of significant differences: smokers exhibited markedly elevated loads as opposed to non-smokers for *Porphyromonas gingivalis* (W = −5.18, *p* < 0.001), *Tannerella forsythensis* (W = −6.40, *p* < 0.001), *Treponema denticola* (W = −4.22, *p* = 0.008), *Fusobacterium nucleatum* (W = −4.540, *p* = 0.004), *Peptostreptococcus micros* (W = −3.86, *p* = 0.018), *Campylobacter gracilis* (W = −3.51, *p* = 0.035), *Campylobacter rectus/showae* (W = −3.954, *p* = 0.014), and *Eubacterium nodatum* (W = −4.22, *p* = 0.008).

E-cigarette users exhibited markedly elevated levels of *Aggregatibacter actinomycetemcomitans* (W = −3.76, *p* = 0.022), *Tannerella forsythensis* (W = −3.91, *p* = 0.016), *Treponema denticola* (W = −3.56, *p* = 0.032), and *Eubacterium nodatum* (W = −3.78, *p* = 0.021) in contrast to non-smokers. *Campylobacter concisus* was considerably more prevalent among non-smokers as opposed to vape users (W = 3.338, *p* = 0.048).

The two exposed groups exhibited significant differences in two species, *Porphyromonas gingivalis* (W = 3.89, *p* = 0.017) and *Campylobacter rectus/showae* (W = 3.857, *p* = 0.018), both of which were elevated in smokers compared to vape users (Table 3). Given the limited sample size, the absence of statistically significant differences for the remaining species should not be interpreted as evidence that subgingival bacterial loads are equivalent between smokers and e-cigarette users.

## 4. Discussion

Our study supports the hypothesis that cigarette smoking and vaping might increase the level of certain periopathogens as compared to non-smokers. Subgingival dental plaque in classic cigarette smokers has a high prevalence of *Porphyromonas gingivalis* (was detected in 94.4% of smokers), *Tannerella forsythia* (100%), and *Treponema denticola* (77.8% of smokers).

Subgingival dental plaque in vapers has a high prevalence of *Porphyromonas gingivalis* (75.0% of e-cigarette users), *Tannerella forsythia* (87.5% of vapers), and *Treponema denticola* (81.2% of vapers). Also, non-smokers had increased amounts of the following periopathogens: *Prevotella nigrescens* was carried by 87.5% of non-smokers, with *Streptococcus gordonii* being detected in 75.0% of non-smokers.

Alblowi found that the heavy-smoker group had elevated abundances of *Fusobacterium nucleatum*, *Veillonella alcalescens*, and *Prevotella oris* at the species level. The intermediate-smoker group exhibited elevated levels of *Veillonella alkalescens* and *Prevotella oris*, succeeded by *Dialister pneumosintes* and *Veillonella rogasae*. Ultimately, the non-smoker cohort demonstrated markedly elevated proportions of all *Veillonella* species: *V. dispar*, *V. alcalescens*, *V. atypica*, *V. rogosae*, and *V. parvula* [21].

In a study conducted by Shchipkova et al., active smokers had elevated levels of *Treponema socranskii* (*p* < 0.05), *Dialister pneumosintes* (*p* < 0.01), and *Peptostreptococcus* sp. oral clones BS044, FG014, AP24, and 2002–69396 97 (*p* < 0.05) [22].

Tamashiro et al. found that for smokers, as the reference group, the plaque index (b  =  0.5682, *p*  =  0.006), smoking status (b  =  −3.83, *p*  =  0.005), and the interaction between smoking status and probing depth (b  =  0.970, *p*  <  0.001) emerged as significant predictors of phylogenetic diversity, whereas probing depth alone and the interaction between smoking status and plaque index did not demonstrate significance [23].

Camelo-Castillo et al. [24] found that bacterial variety was greater in periodontal patients compared to healthy individuals, potentially reflecting a more nutritionally abundant environment or diminished immunological competence. Moreover, smoking affects periodontal ecology.

Several of our significant findings align closely with previously published work. In a study by Aldakheel et al., *A. actinomycetemcomitans* (*p* < 0.001) and *P. gingivalis* (*p* < 0.001) were considerably elevated in cigarette smokers (*p* < 0.01) and users of electronic nicotine delivery systems (*p* < 0.01) compared to non-smokers with periodontitis [25].

The prevalence of Porphyromonas and Veillonella (*p* = 0.008) was greater among vapers in a study by Pushalkar et al., and interleukin (IL)-6 and IL-1β levels were significantly raised in e-cigarette users compared to non-users [26]. Our data diverge from the existing literature with the levels of *Veillonella parvula* being greater in non-smokers compared to vape users.

Our findings regarding the elevated levels of *Porphyromonas gingivalis* in vape users correspond closely with Xu et al.’s findings, which found that e-cigarette use may similarly affect the bacterial composition of saliva over time like cigarette smoking, resulting in an increased prevalence of periodontal disease-related pathogens such as *Porphyromonas gingivalis* and *Fusobacterium nucleatum* [27].

*Porphyromonas gingivalis* was positively identified in the saliva of both electronic cigarette users (*n* = 1) and nicotine pouch users (*n* = 2), while *Prevotella intermedia* was also positively identified in the saliva of electronic cigarette users (*n* = 1) and nicotine pouch users (*n* = 2) in a study conducted by Miluna-Meldere et al. [28].

E-cigarette vapor, irrespective of nicotine concentration, caused DNA damage in oral epithelial cells, accompanied by reduced proliferation and cell survival in a study done by Cátala-Valentín et al. [29].

Jeong et al. found that periodontal disease exhibited greater prevalence among both vapers and smokers compared to non-users in men (electronic cigarettes: odds ratio [OR] = 2.34, 95% confidence interval [CI] = 1.52 to 3.59; conventional cigarettes: OR = 2.17, 95% CI = 1.76 to 2.68) [30].

In comparison to smokers and controls, the metagenome of e-cigarette users exhibited increased abundances of genes associated with ABC transporters and RNA processing and modification systems, alongside virulence factors including cell wall and capsular polysaccharides, biosynthesis of peptidoglycan and lipopolysaccharides, stress response mechanisms, quorum sensing, biofilm formation, resistance to antibiotics and toxic substances, flagellar motility, and siderophores [31].

In study by Pandarathodiyil et al., it was indicated that e-cigarettes emit fewer pollutants and carcinogens compared to traditional cigarettes; however, it also showed markedly elevated salivary LDH levels among vapers [32].

In a study by Yang et al. [33], data from 18 vapers and 18 non-vapers were collected. Nearly 56% of vapers also engaged in smoking traditional cigarettes. Disparities in beta diversity were seen between vapers and non-vapers. Vapers exhibited a markedly greater relative abundance of an unidentified species of Veillonella in comparison to non-vapers.

Smokers exhibited increased quantities of anaerobes and diminished levels of aerobes relative to non-smokers (*p* = 0.02, *t*-test) in a study conducted by Mason et al. [34]. The subgingival microbial composition of smokers exhibited an enrichment of periodontal and systemic pathogens such as *Fusobacterium nucleatum*, *F. naviforme*, *Filifactor alocis*, *Dialister microaerophilus*, *Desulfobulbus* sp. clone R004, *Megasphaera sueciensis*, *M. geminatus*, *M. elsdenii*, *M. micronuciformis*, *Acinetobacter johnsonii*, *A. guillouiae*, *A. schindleri*, *A. baumannii*, *A. haemolyticus*, *Pseudomonas pseudoalcaligenes*, and *Pseudoramibacter alactolyticus*.

Griffen et al. sequenced 454 16S rRNA genes to analyze subgingival bacterial populations from 29 periodontally healthy individuals and 29 participants with chronic periodontitis [35]. Variations in bacterial communities associated with health and periodontitis were noted at every phylogenetic tier, and UniFrac and principal coordinate analyses revealed unique community patterns in health versus disease. Disease exhibited more community variety, with 123 species identified as considerably more abundant in diseased and 53 in healthy individuals. *Spirochaetes*, *Synergistetes*, and *Bacteroidetes* exhibited greater abundance in sick individuals, while Proteobacteria were present in elevated quantities in healthy controls. In the phylum Firmicutes, the class Bacilli is linked to health, while *Clostridia*, *Negativicutes*, and *Erysipelotrichia* are linked to disease [35].

Samples of marginal and subgingival plaques, as well as gingival crevicular fluid, were obtained from 15 current smokers and 15 never-smokers after 1, 2, 4, and 7 days of uninterrupted plaque accumulation in a study by Kumar et al. [36]. Cloning and sequencing of the 16S rRNA gene facilitated bacterial identification, while multiplex bead-based flow cytometry quantified the concentrations of 27 immunological mediators. Smokers exhibited a markedly varied and rather unstable initial colonization of both marginal and subgingival biofilms, characterized by reduced niche saturation compared to non-smokers. Periodontal pathogens from the genera *Fusobacterium*, *Cardiobacterium*, *Synergistes*, and *Selenomonas*, along with respiratory pathogens from the genera *Haemophilus* and Pseudomonas, colonized the initial biofilms of smokers and persisted throughout the observation period, indicating that smoking promotes the early acquisition and colonization of pathogens in oral biofilms. Smokers had an early proinflammatory response to this colonization that lasted for over 7 days. A favorable link between proinflammatory cytokine levels and commensal bacteria was identified in smokers, but not in non-smokers [36].

In periodontal illnesses, the subgingival microbiota of smokers exhibits a pathogen-dominant community with diminished resistance compared to non-smokers, complicating treatment efforts in a study by Jiang et al. [37]. Biological modifications in critical pathogens, such as *Porphyromonas gingivalis*, coupled with an inadequate host immune response for its removal, may lead to changes in the subgingival microbiota of smokers [37].

The microbiome of electronic cigarette users exhibited numerous similarities with that of traditional smokers and certain aspects with non-smokers, while also possessing a distinct subgingival microbial community enriched in *Fusobacterium* and *Bacteroidales* in a study by Thomas et al. [38]. The data indicate that the use of electronic cigarettes fosters a distinct periodontal microbiota, maintaining a stable heterogeneous condition between traditional smokers and non-smokers, and posing unique oral health concerns [38]. In our study, *Fusobacterium nucleatum* was present in 87.5% of non-smokers and in 100% of smokers.

Tobacco smoking markedly modifies the bacterial profiles in fecal, buccal, and salivary samples. In comparison to the controls, exposure to electronic cigarettes did not influence the oral or gut microbiomes in a study by Stewart et al. [39]. The alterations in the gut microbiota of tobacco users correlated with an elevated relative abundance of *Prevotella* and a diminished relative abundance of Bacteroides [39].

The saliva of e-cigarette users exhibited alterations in antibacterial characteristics when compared to both the control group and traditional cigarette smokers in a study by Cichońska et al. [40].

Charde et al. found that vaping may correlate with increased clinical attachment loss in contrast to non-smokers and adversely affect periodontal bacteria counts, inflammatory biomarkers, and oxidative stress levels [41].

The periodontal condition is inferior, and levels of proinflammatory cytokines in gingival crevicular fluid are elevated in cigarette smokers compared to electronic cigarette smokers and non-smokers in a study by BinShabaib et al. [42].

An increase in Streptococcus mutans proliferation was noted in e-cigarette users, particularly during the initial culture phase in a study by Rouabhia et al. [43].

The observed differences in OHI-S scores across the three study groups suggest that both conventional cigarette smoking and e-cigarette use are associated with a comparatively lower standard of oral hygiene relative to non-smoking individuals, despite the periodontally healthy status of all participants. While non-smokers maintained a mean score within the good oral hygiene category, both smoker and e-cigarette user groups shifted into the fair oral hygiene range.

This pattern is consistent with previous reports linking elevated OHI-S values to suboptimal oral hygiene status in populations exposed to tobacco- and nicotine-containing products. Poor oral hygiene, as reflected by the OHI-S score, was observed in 70% of tobacco users compared with 28% of non-tobacco users (*p* < 0.05) in a study by Mahajan et al. [44].

Substandard oral health was more common than satisfactory oral health among daily e-cigarette users (55.5% compared 44.5%, *p* < 0.0001) and intermittent e-cigarette users (56% versus 44%, *p* < 0.0001) in a study by Huilgol et al. [45].

This study investigated the subgingival microbiome of young adult cigarette smokers, e-cigarette users, and non-smokers, revealing that both types of smoking were linked to a microbial community richer in known periodontal pathogens. The most reliable indicator pertained to the red-complex bacteria—*Porphyromonas gingivalis*, *Tannerella forsythia*, and *Treponema denticola*—which were present more frequently and in greater quantities among smokers and vapers compared to non-smokers.

From a clinical and public-health perspective, these results are relevant because they concern young adults—a population in which e-cigarette use is widespread and frequently regarded as harmless. The presence of a periodontopathogen-enriched subgingival microbiome at this age may represent an early, modifiable step. Also, higher concentrations of periopathogens represent a great concern regarding the future evolution to severe or early-onset periodontitis.

From a clinical perspective, the increased detection frequency of red-complex bacteria (*P. gingivalis*, *T. forsythia*, and *T. denticola*) observed in both cigarette smokers and e-cigarette users, despite the absence of clinical signs of periodontal disease, suggests that subclinical colonization by periodontal pathogens may precede overt clinical manifestations in this population. This finding raises the possibility that smokers and e-cigarette users who present as periodontally healthy at clinical examination may nonetheless harbor a microbiological profile associated with elevated future risks. However, given the cross-sectional design of this study, these findings should be interpreted with caution: they cannot establish a causal relationship between e-cigarette or cigarette use and periodontal pathogen colonization, nor can they predict whether this subclinical bacterial shift will translate into a clinically significant periodontal breakdown over time.

This study has several limitations due to its relatively small group sizes that limit the results, and the fact that, due to the young age of the sample, smoking and vaping habits did not last for a long period in some patients. That is why we established as an inclusion criterion a minimum of 3 years of smoking/vaping.

Longitudinal studies are needed to determine whether the microbiological differences observed here precede, and are predictive of, future periodontal disease progression, before firm clinical recommendations can be made. The relatively small sample size, determined by feasibility rather than a priori power calculation, limits the statistical power to detect smaller effect sizes and restricts the generalizability of the findings. Larger, adequately powered studies are needed to confirm these preliminary observations.

## 5. Conclusions

Within the limits of this exploratory, cross-sectional study, both cigarette smoking and e-cigarette use were associated with a higher prevalence of several periodontal pathogens compared with non-smokers, supporting the hypothesis that tobacco and vaping exposure may contribute to subgingival dysbiosis. When analyzing the two exposed groups directly, statistically significant differences were observed for a limited number of species, while the majority of species showed no significant difference between smokers and vapers. Larger, adequately powered, and longitudinal studies are needed to determine whether these similarities reflect a genuine overlap in subgingival microbial risk between the two forms of exposure, or whether they result from limited statistical power in the present sample.

## Figures and Tables

**Table 1:** Presence of each subgingival species in the three groups, with Fisher’s exact test and Cramér’s V. Data are presented as number of carriers (percentage).

Species	Smokers (*n* = 18) *n* (%)	Non-Smokers (*n* = 16) *n* (%)	Vape Users (*n* = 16) *n* (%)	*p* (Fisher)	Cramér’s V
*Aggregatibacter actinomycetemcomitans* (A.a)	3 (16.7)	0 (0.0)	6 (37.5)	**0.018**	0.391
*Porphyromonas gingivalis* (P.g)	17 (94.4)	9 (56.2)	12 (75.0)	**0.028**	0.368
*Tannerella forsythensis* (T.f)	18 (100.0)	11 (68.8)	14 (87.5)	**0.022**	0.372
*Treponema denticola* (T.d)	14 (77.8)	7 (43.8)	13 (81.2)	0.056	0.358
*Prevotella intermedia* (P.i)	10 (55.6)	7 (43.8)	9 (56.2)	0.823	0.113
*Fusobacterium nucleatum* (F.n)	18 (100.0)	14 (87.5)	14 (87.5)	0.370	0.221
*Peptostreptococcus micros* (P.m)	18 (100.0)	15 (93.8)	15 (93.8)	0.530	0.153
*Prevotella nigrescens* (P.n)	9 (50.0)	14 (87.5)	7 (43.8)	**0.022**	0.389
*Campylobacter gracilis* (C.g)	13 (72.2)	5 (31.2)	8 (50.0)	0.063	0.339
*Campylobacter rectus/showae* (C.r/C.sh)	17 (94.4)	15 (93.8)	14 (87.5)	0.830	0.114
*Eubacterium nodatum* (E.n)	15 (83.3)	6 (37.5)	13 (81.2)	**0.012**	0.449
*Eikenella corrodens* (E.c)	17 (94.4)	14 (87.5)	14 (87.5)	0.723	0.111
*Capnocytophaga* species (C.s)	5 (27.8)	9 (56.2)	3 (18.8)	0.086	0.332
*Campylobacter concisus* (C.c)	13 (72.2)	12 (75.0)	7 (43.8)	0.149	0.290
*Streptococcus mitis group* (S.m.g)	14 (77.8)	14 (87.5)	14 (87.5)	0.706	0.127
*Streptococcus gordonii* (S.g)	7 (38.9)	12 (75.0)	11 (68.8)	0.067	0.327
*Streptococcus constellatus* (S.c)	17 (94.4)	13 (81.2)	14 (87.5)	0.492	0.167
*Actinomyces odontolyticus* (A.o)	16 (88.9)	16 (100.0)	16 (100.0)	0.321	0.272
*Actinomyces viscosus* (A.v)	17 (94.4)	16 (100.0)	14 (87.5)	0.515	0.211
*Veillonella parvula* (V.p)	17 (94.4)	15 (93.8)	14 (87.5)	0.830	0.114

Data are *n* (%). *p* values are from Fisher’s exact test; values < 0.05 are shown in bold. Cramér’s V expresses the strength of the association. *n* = 50 (smokers: 18, non-smokers: 16, and vape users: 16).

**Table 2:** Comparison of subgingival bacterial load between the three groups (Kruskal–Wallis test).

Species	χ^2^	df	*p*
*Aggregatibacter actinomycetemcomitans* (A.a)	7.745	2	**0.021**
*Porphyromonas gingivalis* (P.g)	15.581	2	**<0.001**
*Tannerella forsythensis* (T.f)	20.062	2	**<0.001**
*Treponema denticola* (T.d)	11.259	2	**0.004**
*Prevotella intermedia* (P.i)	1.457	2	0.483
*Fusobacterium nucleatum* (F.n)	10.648	2	**0.005**
*Peptostreptococcus micros* (P.m)	7.420	2	**0.024**
*Prevotella nigrescens* (P.n)	3.234	2	0.198
*Campylobacter gracilis* (C.g)	6.042	2	**0.049**
*Campylobacter rectus/showae* (C.r/C.sh)	10.589	2	**0.005**
*Eubacterium nodatum* (E.n)	10.741	2	**0.005**
*Eikenella corrodens* (E.c)	3.494	2	0.174
*Capnocytophaga* species (C.s)	4.353	2	0.113
*Campylobacter concisus* (C.c)	6.865	2	**0.032**
*Streptococcus mitis group* (S.m.g)	1.085	2	0.581
*Streptococcus gordonii* (S.g)	5.908	2	0.052
*Streptococcus constellatus* (S.c)	2.526	2	0.283
*Actinomyces odontolyticus* (A.o)	0.685	2	0.710
*Actinomyces viscosus* (A.v)	4.952	2	0.084
*Veillonella parvula* (V.p)	1.286	2	0.526

χ^2^ = Kruskal–Wallis H statistic; df = degrees of freedom; *p* values < 0.05 are shown in bold. Statistically significant omnibus results were followed by Dwass–Steel–Critchlow–Fligner pairwise comparisons.

**Table 3:** Dwass–Steel–Critchlow–Fligner (DSCF) pairwise comparisons of subgingival bacterial load between the three groups (vape users, smokers, and non-smokers) for all 20 species.

Species	Vape vs. Smoker		Vape vs. Non-Smoker		Smoker vs. Non-Smoker	
	W	*p*	W	*p*	W	*p*
*Aggregatibacter actinomycetemcomitans* (A.a)	−2.07	0.307	−3.76	**0.022**	−2.38	0.212
*Porphyromonas gingivalis* (P.g)	3.89	**0.017**	−1.93	0.359	−5.18	**<0.001**
*Tannerella forsythensis* (T.f)	1.81	0.408	−3.91	**0.016**	−6.40	**<0.001**
*Treponema denticola* (T.d)	2.11	0.296	−3.56	**0.032**	−4.22	**0.008**
*Prevotella intermedia* (P.i)	0.281	0.979	−1.310	0.624	−1.617	0.488
*Fusobacterium nucleatum* (F.n)	3.222	0.059	−0.507	0.932	−4.540	**0.004**
*Peptostreptococcus micros* (P.m)	1.76	0.428	−1.97	0.344	−3.86	**0.018**
*Prevotella nigrescens* (P.n)	1.454	0.559	2.884	0.103	0.248	0.983
*Campylobacter gracilis* (C.g)	1.46	0.558	−1.89	0.376	−3.51	**0.035**
*Campylobacter rectus/showae* (C.r/C.sh)	3.857	**0.018**	0.827	0.828	−3.954	**0.014**
*Eubacterium nodatum* (E.n)	1.03	0.748	−3.78	**0.021**	−4.22	**0.008**
*Eikenella corrodens* (E.c)	1.05	0.738	−1.36	0.600	−2.71	0.134
*Capnocytophaga* species (C.s)	1.21	0.667	3.16	0.066	1.28	0.635
*Campylobacter concisus* (C.c)	3.113	0.071	3.338	**0.048**	0.517	0.929
*Streptococcus mitis group* (S.m.g)	−0.587	0.910	0.747	0.858	1.517	0.531
*Streptococcus gordonii* (S.g)	−2.887	0.103	0.297	0.976	3.062	0.077
*Streptococcus constellatus* (S.c)	1.416	0.576	−0.748	0.857	−2.198	0.266
*Actinomyces odontolyticus* (A.o)	0.391	0.959	−0.533	0.925	−1.270	0.642
*Actinomyces viscosus* (A.v)	2.709	0.134	2.719	0.132	0.390	0.959
*Veillonella parvula* (V.p)	1.587	0.501	0.587	0.910	−0.952	0.779

W = Dwass–Steel–Critchlow–Fligner test statistic; *p*-values < 0.05 are shown in bold. Comparisons are post hoc tests carried out after a significant Kruskal–Wallis test and refer to differences in bacterial load between the paired groups.

## Data Availability

The data supporting the findings of this study are available from the corresponding authors upon reasonable request. The data are not publicly available due to ethical and privacy restrictions.

## Abbreviations

The following abbreviations are used in this manuscript:

A.a	*Aggregatibacter actinomycetemcomitans*
A.o	*Actinomyces odontolyticus*
A.v	*Actinomyces viscosus*
BOP	Bleeding on probing
C.c	*Campylobacter concisus*
C.g	*Campylobacter gracilis*
C.r/C.sh	*Campylobacter rectus/showae*
C.s	*Capnocytophaga* species
E.c	*Eikenella corrodens*
E.n	*Eubacterium nodatum*
F.n	*Fusobacterium nucleatum*
OHI-S	Simplified Oral Hygiene Index
PCR	Polymerase Chain Reaction
PD	Probing depth
P.g	*Porphyromonas gingivalis*
P.i	*Prevotella intermedia*
P.m	*Peptostreptococcus micros*
P.n	*Prevotella nigrescens*
S.c	*Streptococcus constellatus*
S.g	*Streptococcus gordonii*
S.m.g	*Streptococcus mitis group*
T.d	*Treponema denticola*
T.f	*Tannerella forsythensis*
V.p	*Veillonella parvula*
